# Role of olfactory receptor Olfr544 in neuronal cells: Insights into neurite outgrowth and CREB signaling

**DOI:** 10.1016/j.isci.2026.117757

**Published:** 2026-10-09

**Authors:** Shuhan Lv, Jia Liu, Yiyuan Zhou, Rong Yang, Taiwei Zhang, Yunyan Deng, Yan Chen, Chencen Lai, Qian Chen

**Affiliations:** 1 Research Center of Eugenics, The First Affiliated Hospital of Guizhou University of Traditional Chinese Medicine, Guiyang, Guizhou, China; 2 Department of Obstetrics, The First Affiliated Hospital of Guizhou University of Traditional Chinese Medicine, Guiyang, China; 3 College of First Clinical Medicine, Guizhou University of Traditional Chinese Medicine, Guiyang, Guizhou, China; 4 Guizhou Detianyue Trading Co., Ltd., Guiyang, Guizhou, China; 5 Department of Nosocomial Infection, The First Affiliated Hospital of Guizhou University of Traditional Chinese Medicine, Guiyang, China

**Keywords:** olfactory receptor Olfr544, neurite outgrowth, CREB signaling, neuronal cells, neurodegenerative diseases

## Abstract

Olfactory receptors (ORs) are expressed beyond the nasal epithelium and implicated in brain function, yet their roles remain unclear. This study investigated the function of the OR Olfr544, which has been reported to be highly expressed near amyloid plaques in Alzheimer’s disease model mice, using the mouse neuroblastoma N2a cell line. We found that its agonist azelaic acid (AZA) promotes neurite outgrowth and activates the cAMP-PKA-CREB signaling cascade in an Olfr544-dependent manner. Transcriptome analysis of *Olfr544*-knockout cells revealed altered expression of genes involved in neuroactive signaling and cytoskeletal organization. Key downstream targets, including Igf2bp3, were identified and validated. These results establish a functional link between an extra-olfactory OR and neuronal morphology via CREB *in vitro*, providing a mechanistic basis for further studies of OR functions in the nervous system.

## Highlights

• Olfr544 activation by AZA promotes neurite outgrowth in N2a cells via CREB signaling
• Olfr544 couples to a canonical cAMP-PKA-CREB cascade in neuronal cells
• *Olfr544* knockout alters neuroactive signaling and cytoskeletal gene expression
• IGF2BP3, SORBS2, and KCTD14 are identified as CREB targets downstream of Olfr544

## Graphical abstract

**Figure:**
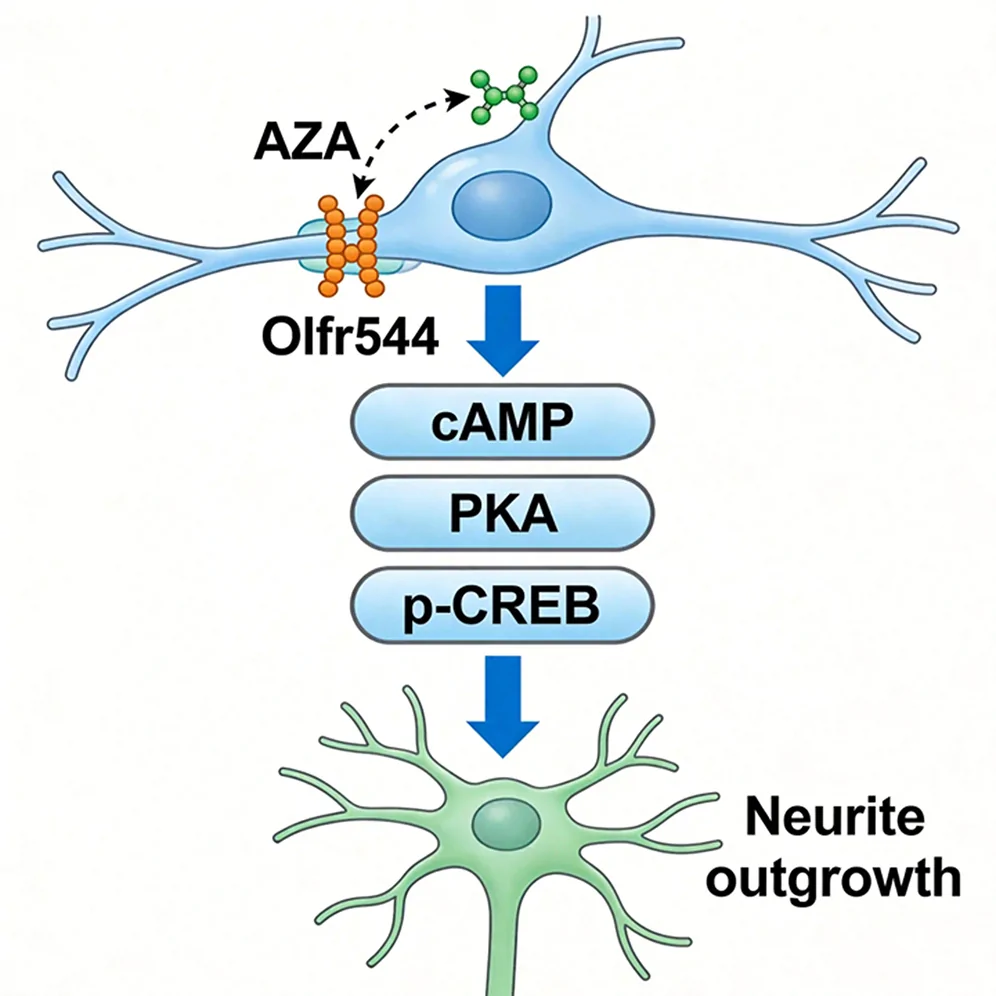

## Teaser

Biological sciences; Molecular neuroscience; Natural sciences; Neuroscience

## Introduction

Olfactory receptors (ORs) are critical components of the animal olfactory system.^1^ However, following their initial discovery in the olfactory epithelium, ORs were found to be widely expressed in various non-olfactory tissues, where they exert important biological functions.^2^^,^^3^^,^^4^ Recently, the expression of ORs and their associated downstream molecules (AC3 and Gαolf) has been detected in multiple regions of the human, mouse, and rat brain.^5^ This finding indicates that ORs expressed in the brain may function as G protein-coupled receptors (GPCRs). Such extra-olfactory expression raises important questions regarding their physiological roles within the central nervous system (CNS).

Emerging evidence suggests that ORs may be involved in crucial neural processes. Specifically, certain brain-expressed ORs have been shown to modulate microglial activation and phagocytosis or influence tau phosphorylation, suggesting their direct involvement in brain pathophysiology.^6^^,^^7^ Particularly compelling is the association between specific ORs and neurodegenerative diseases such as Alzheimer’s disease (AD). For instance, the OR Olfr544 has been found to be highly expressed in the cerebral cortex and the hippocampus of 9-month-old 5×FAD transgenic mice, an AD model. Its expression localizes near Aβ plaques, a key pathological hallmark, and varies with age and disease progression. Furthermore, the mRNA level of *Olfr544* exhibited significant variations correlated with mouse age and pathology.^8^

The activator of Olfr544 is azelaic acid (AZA).^9^ As one of the relatively few deorphanized ORs, the functions of Olfr544 in the pancreas and liver have been elucidated.^10^^,^^11^^,^^12^ However, the functional role of Olfr544, which is highly expressed in the brains of AD mice, remains unknown within the CNS.

Given the importance of CREB signaling in neuronal survival, plasticity, and memory formation, and considering that ORs can couple to canonical GPCR pathways that modulate intracellular cAMP and Ca^2+^ levels, we hypothesized that Olfr544 might regulate neuronal morphology and function via CREB-dependent mechanisms. Testing this hypothesis is crucial not only for understanding the non-olfactory functions of ORs in the brain but also for evaluating their potential as therapeutic targets in neurodegenerative disorders.

Therefore, we utilized the mouse neuroblastoma N2a cell line to dissect the neuronal function of OR Olfr544. We first verified endogenous expression of the complete Olfr544-Gαolf-AC3 signaling axis in N2a cells and confirmed that its agonist AZA facilitates neurite outgrowth in an Olfr544-dependent manner. Heterologous reconstitution in Olfr544-null HEK293T cells further validated the direct Olfr544-CREB transcriptional axis. Biochemical assays demonstrated that Olfr544 activation triggers the canonical cAMP-PKA-CREB signaling cascade, while CRISPR-Cas9-mediated *Olfr544* knockout (KO) fully abolished AZA-induced elevation of intracellular cAMP, Protein Kinase A (PKA) activity, and phosphorylated CREB (pCREB). Transcriptome profiling of *Olfr544*-KO cells uncovered altered transcriptional programs related to neuroactive ligand signaling and cytoskeletal motor proteins. We further predicted and experimentally validated three high-confidence CREB downstream targets, including Igf2bp3, Sorbs2, and Kctd14, with IGF2BP3 showing the most robust Olfr544-dependent regulation. Collectively, this work aims to clarify how extra-olfactory Olfr544 regulates neuronal morphology via CREB signaling, offering mechanistic insights into the neurobiological functions of extra-olfactory ORs.

## Results

### Endogenous expression of Olfr544 signaling components in neuronal N2a cells

To establish a model for studying Olfr544 function in a neuronal context, we first confirmed the expression of the receptor and its canonical downstream signaling molecules in the mouse neuroblastoma N2a cell line. Total RNA was extracted from N2a cells and reverse-transcribed into cDNA. Specific primers for *Olfr544*, *Gαolf*, and *AC3* were designed on the basis of sequence information obtained from NCBI. PCR amplification with these primers produced specific products, which were confirmed as *Olfr544*, *Gαolf*, and *AC3* through agarose gel electrophoresis and sequencing (Figure 1A). This confirmed that N2a cells endogenously express a functional Olfr544 signaling axis, providing a relevant cellular system for subsequent functional investigations.

**Figure 1:**
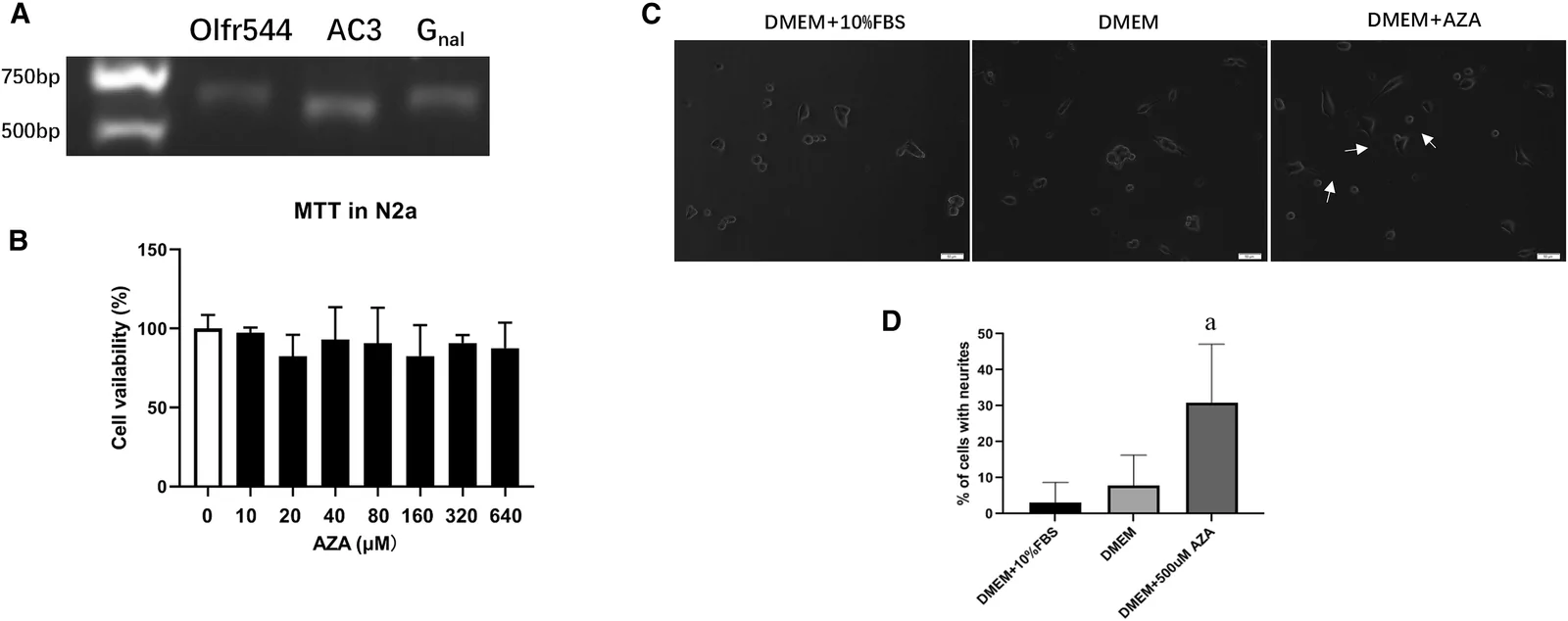
Endogenous *Olfr544* signaling components and agonist-dependent neurite outgrowth in N2a neuronal cells (A) Agarose gel electrophoresis of RT-PCR products demonstrating endogenous expression of *Olfr544*, *Gαolf*, and *AC3*. (B) Effect of varying concentrations of AZA on N2a cell viability assessed by MTT assay. *n* = 6 biologically independent samples per group. (C) Phase-contrast microscopy images showing AZA-induced neurite outgrowth in N2a cells (scale bars, 50 μm). (D) Quantification of the percentage of cells exhibiting neurite outgrowth relative to total cells. Data represent mean ± SEM; (A) *p* < 0.05 compared to wild type; *n* = 3 biologically independent experiments, with 10 fields of view quantified per experiment.

### AZA, the Olfr544 agonist, promotes neurite outgrowth in N2a cells

Prior to assessing the functional effects of Olfr544 activation, we determined a non-cytotoxic concentration range for its specific agonist, AZA. An MTT (3-(4,5-dimethylthiazol-2-yl)-2,5-diphenyltetrazolium bromide) assay revealed that N2a cell viability was unaffected by AZA concentrations up to 640 μM (Figure 1B), indicating that any observed phenotypic changes within this range are unlikely to result from altered cell survival. Consistent with our findings, literature reports have also shown that AZA concentrations ranging from 0 to 500 μM exert no significant effect on the viability of adipocyte-derived 3T3-L1 cells and hepatoma-derived Hepa1c1c-7 cells.

We next asked whether Olfr544 activation influences neuronal morphology, a key aspect of neuronal differentiation and plasticity. N2a cells were treated with 500 μM AZA in serum-deprived DMEM for 24 h. Cell morphology was then examined and imaged with phase-contrast microscopy. For each sample, images were captured from 10 randomly selected fields of view. The number of cells exhibiting significant process outgrowth (defined as processes longer than the cell body diameter) was quantified and counted with ImageJ software, and the percentage of such cells among the total cell population was calculated. The differences were statistically analyzed. Results demonstrated that treatment with 500 μM AZA for 24 h induced process outgrowth in approximately 30% of N2a cells (Figures 1C and 1D). The 500 μM concentration for this 24-h morphological assay was selected from independent dose-response pre-experiments (50–500 μM), as it produced the most robust and consistent neurite-promoting effect while remaining well within the non-cytotoxic range determined by the MTT assay (Figure 1B). This represented a statistically significant increase compared to serum-deprived control cultures, where only about 10% of the cells displayed process outgrowth—a baseline level consistent with the neurite-promoting effect of short-term serum deprivation reported in the literature. This result suggested that Olfr544 signaling may play a role in regulating neurite extension. To rigorously test whether this effect was specifically mediated by Olfr544, a genetic approach was required. However, direct morphological comparison by using *Olfr544*-KO cells under the same differentiation conditions was confounded by their intrinsically poorer baseline growth and adhesion (noted in discussion). Therefore, to definitively establish the receptor’s essential role, we employed genetic KO and focused subsequent validation on downstream signaling and transcriptional outputs.

### Heterologous reconstitution validates the direct Olfr544-CREB transcriptional axis

To directly test whether AZA activates CREB signaling specifically through Olfr544, independent of the complex background of neuronal cells, we reconstituted the receptor in a heterologous system. While previous studies utilizing Hana3 cells have well established AZA as a specific agonist for Olfr544, our goal here was to explicitly confirm that this receptor’s activation is sufficient to directly drive CREB-mediated transcription. We cloned the full-length mouse *Olfr544* cDNA into the pME18S-Lucy-Flag-Rho vector, which facilitates proper membrane trafficking of ORs (Figure 2A). This construct was co-transfected into HEK293T cells—which lack endogenous Olfr544—alongside a CREB-responsive dual-luciferase reporter. In cells expressing Olfr544, AZA stimulation significantly induced CREB-driven luciferase activity, while no response was observed in empty-vector transfected cells. Moreover, the AZA-induced CREB activity was markedly reduced when cells were co-treated with the adenylate cyclase inhibitor SQ22536 (Figure 2B), confirming the general functionality of the downstream signaling pathway. This result not only verifies the functionality of our expression construct but also demonstrates that Olfr544 is sufficient to initiate CREB-dependent transcription in an isolated system. This rules out the possibility that AZA-induced CREB activation relies entirely on secondary, indirect pathways unique to the N2a cellular environment, thereby strengthening the mechanistic link between Olfr544 and CREB in our neuronal model.

**Figure 2:**
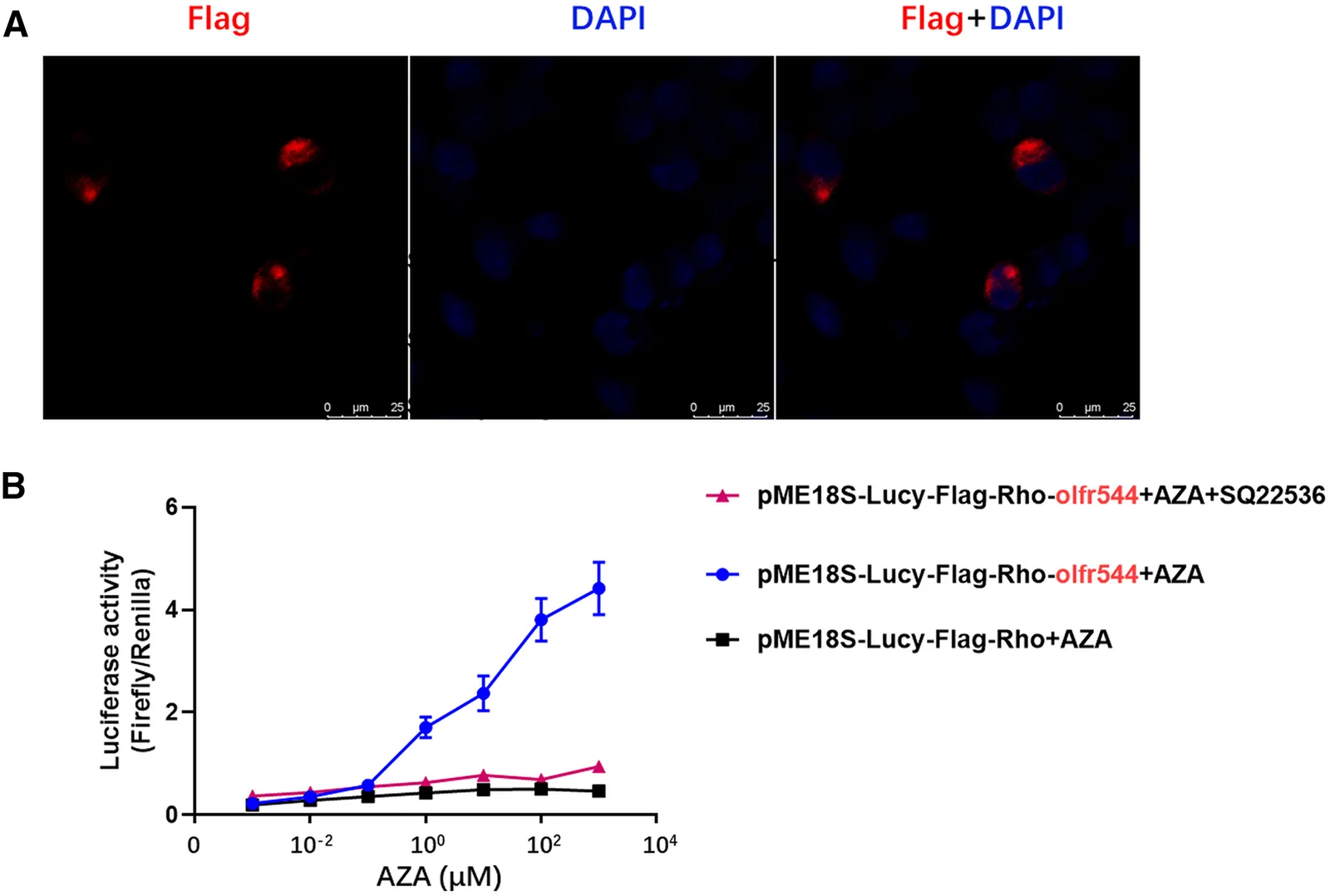
Heterologous expression and functional Analysis of Olfr544 in HEK293T cells (A) Representative confocal microscopy images showing immunofluorescence staining of anti-Flag (red, indicating Olfr544-Flag fusion protein) and DAPI (blue, nuclei). (B) Activation of Olfr544 by AZA assessed through a dual-luciferase reporter assay. *n* = 3 biologically independent experiments.

### Olfr544 couples to a canonical cAMP-PKA-CREB signaling cascade

To genetically dissect the function of Olfr544 in N2a cells, we generated a stable *Olfr544*-KO cell line with CRISPR-Cas9 technology. Two single-guide RNAs (sgRNAs) targeting exon 2 of the mouse *Olfr544* gene were designed and transfected into N2a cells. Following puromycin selection, monoclonal populations were isolated by limited dilution. PCR amplification and Sanger sequencing of the targeted genomic locus confirmed a homozygous 149-bp deletion that introduces a frameshift and a premature stop codon, resulting in the loss of functional *Olfr544* transcript (Figure S2). However, we acknowledge that direct protein-level confirmation of Olfr544 ablation was not achievable due to the lack of a validated specific antibody. This KO cell line was subsequently used for all loss-of-function experiments described in the following text.

CREB is a central transcription factor linking extracellular signals to neuronal gene expression programs involved in growth and plasticity. We hypothesized that Olfr544 activation might transduce its morphological effects through CREB signaling. Similarly, in preliminary experiments with a concentration- and time course matrix, 50 μM AZA for 30 min consistently produced the most stable and reproducible pCREB signal and was, therefore, selected as the standard condition for all subsequent signaling assays. Western blot analysis showed that a 30-min stimulation with 50 μM AZA significantly increased the levels of pCREB in wild-type (WT) N2a cells. Notably, the baseline pCREB level in unstimulated *Olfr544*-KO cells was comparable to that of unstimulated WT cells. Crucially, AZA stimulation failed to induce any increase in pCREB in the KO cells (Figure 3), confirming that AZA-induced CREB activation is strictly dependent on Olfr544. This finding provides direct molecular evidence that Olfr544 functions as a bona fide receptor for AZA in neuronal cells, coupling to intracellular pathways that converge on CREB.

**Figure 3:**
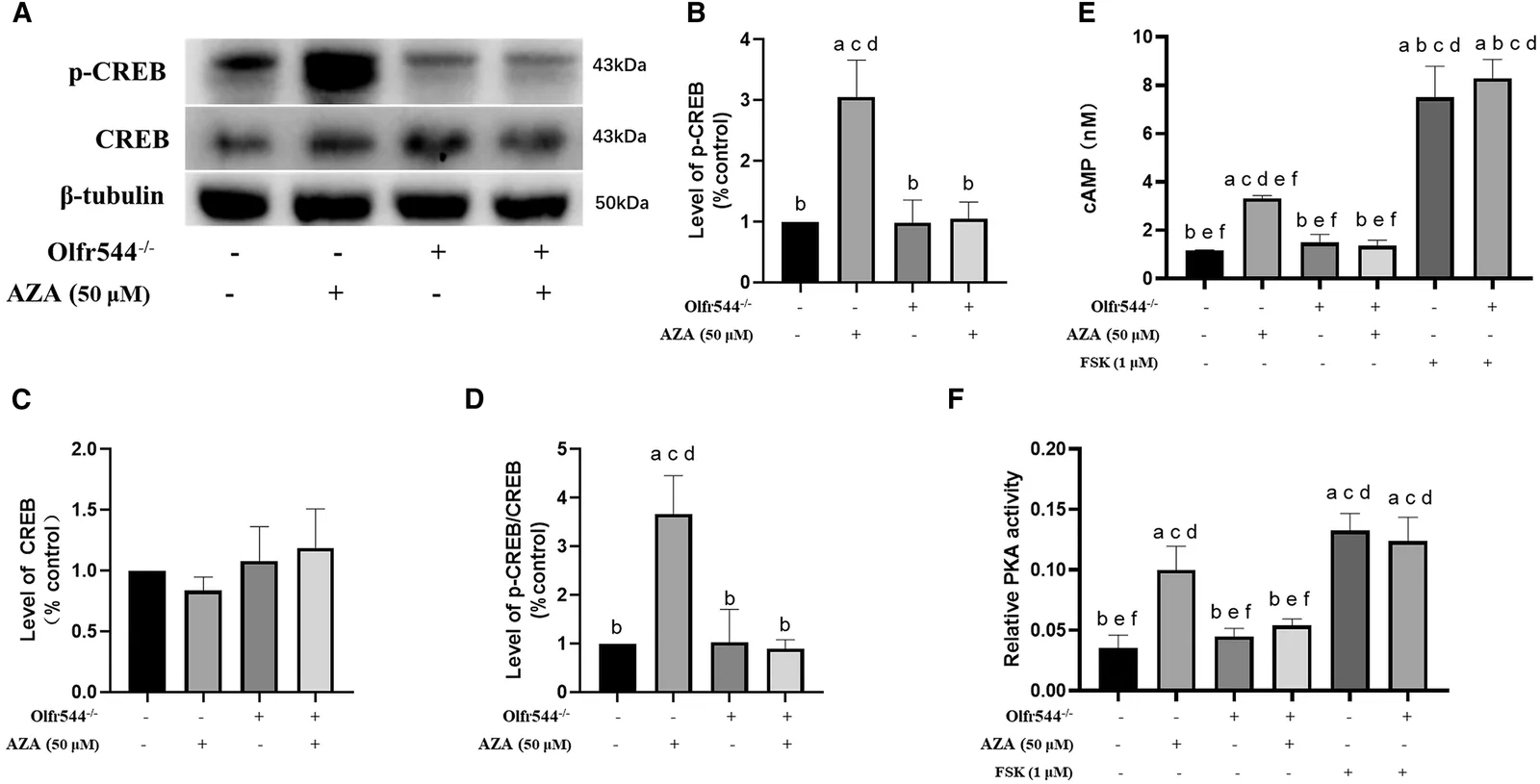
Expression of pCREB and CREB in N2a cells after 30 min of AZA treatment (A) Western blot results. (B–D) Band intensity analysis results with ImageJ. The relative protein expression level in the blank control group was set as 1. (E) Quantification of intracellular cAMP levels in N2a wild-type (WT) and *Olfr544*-knockout (KO) cells treated with AZA (50 μM) or forskolin (FSK, 1 μM) for 30 min, as detected by ELISA. (F) Quantification of relative PKA activity in N2a WT and *Olfr544*-KO cells treated with AZA (50 μM) or FSK (1 μM) for 30 min. Data are from *n* = 3 biologically independent experiments and presented as mean ± SEM. One-way ANOVA followed by Tukey’s HSD (honestly significant difference) test and Student’s *t* test were performed for multiple group comparisons. (A) *p* < 0.05 compared to the control group (untreated WT cells); (B) *p* < 0.05 compared to WT + AZA; (C) *p* < 0.05 compared to KO cell; (D) *p* < 0.05 compared to KO + AZA; (E) *p* < 0.05 compared to WT + FSK; (F) *p* < 0.05 compared to KO + FSK.

Having established that Olfr544 is required for AZA-induced CREB phosphorylation, we next sought to define the upstream signaling pathway linking receptor activation to this key nuclear event. Canonical GPCR signaling often proceeds through Gαs proteins to activate adenylyl cyclase (AC), elevate cAMP, and stimulate PKA, which in turn phosphorylates CREB. To test whether Olfr544 engages this conserved pathway, we measured its proximal components. ELISA revealed that AZA stimulation triggered a rapid and significant increase in intracellular cAMP in WT N2a cells, although the magnitude of this increase was lower than that induced by the direct AC activator forskolin (FSK) (Figure 3E). Crucially, baseline cAMP levels in unstimulated KO cells were similar to those in untreated WT cells, but the cAMP response to AZA was completely abolished in *Olfr544*-KO cells. Consistent with the upstream signal, AZA treatment significantly increased PKA activity in WT, but not in *Olfr544*-KO cells, which maintained basal PKA activity comparable to WT controls (Figure 3F). These results, together with the Olfr544-dependent phosphorylation of CREB (Figure 3), delineate a linear signaling cascade: Olfr544 activation → cAMP elevation → PKA activation → CREB phosphorylation. This mechanistic mapping positions Olfr544 within a classic GPCR framework and explains how its engagement translates into the activation of a central transcription factor governing neuronal plasticity.

### Transcriptomic profiling of an *Olfr544*-KO clone reveals candidate pathways associated with neuronal structure

To gain a broader, hypothesis-generating view of pathways potentially affected by *Olfr544* deletion, we performed RNA sequencing (RNA-seq) on WT N2a cells and a single monoclonal *Olfr544*-KO line. It is important to note that this comparison reflects the transcriptome of one specific CRISPR-Cas9-derived clone and, therefore, captures both the consequences of *Olfr544* loss and potential clonal adaptation artifacts inherent to the single-cell selection process. Consequently, the differential expression results presented here should be interpreted strictly as a resource for identifying candidate pathways rather than as a definitive, high-confidence catalog of *Olfr544*-specific transcriptional targets. Transcriptome sequencing was performed on six samples (*n* = 3 biologically independent cultures per group), yielding a total of 41.55 GB of clean data, with each sample containing at least 6.03 GB. The Q30 base percentage exceeded 96.31% for all samples. Clean reads from each sample were aligned to the designated reference genome, with mapping rates ranging from 86.18% to 95.07%.

A total of 26,893 expressed genes and 82,202 expressed transcripts were detected. Differential gene expression analysis between groups was conducted on the basis of quantified expression levels through DESeq2, with the following filtering thresholds: |log_2_FC| ≥ 2.807 and padj <0.05. This analysis identified 1,727 differentially expressed genes (DEGs), including 1,397 upregulated genes and 330 downregulated genes (Figure 4). Table 1 lists the top 10 most significantly upregulated and downregulated genes in N2a *Olfr544*^−/−^ cells.

**Figure 4:**
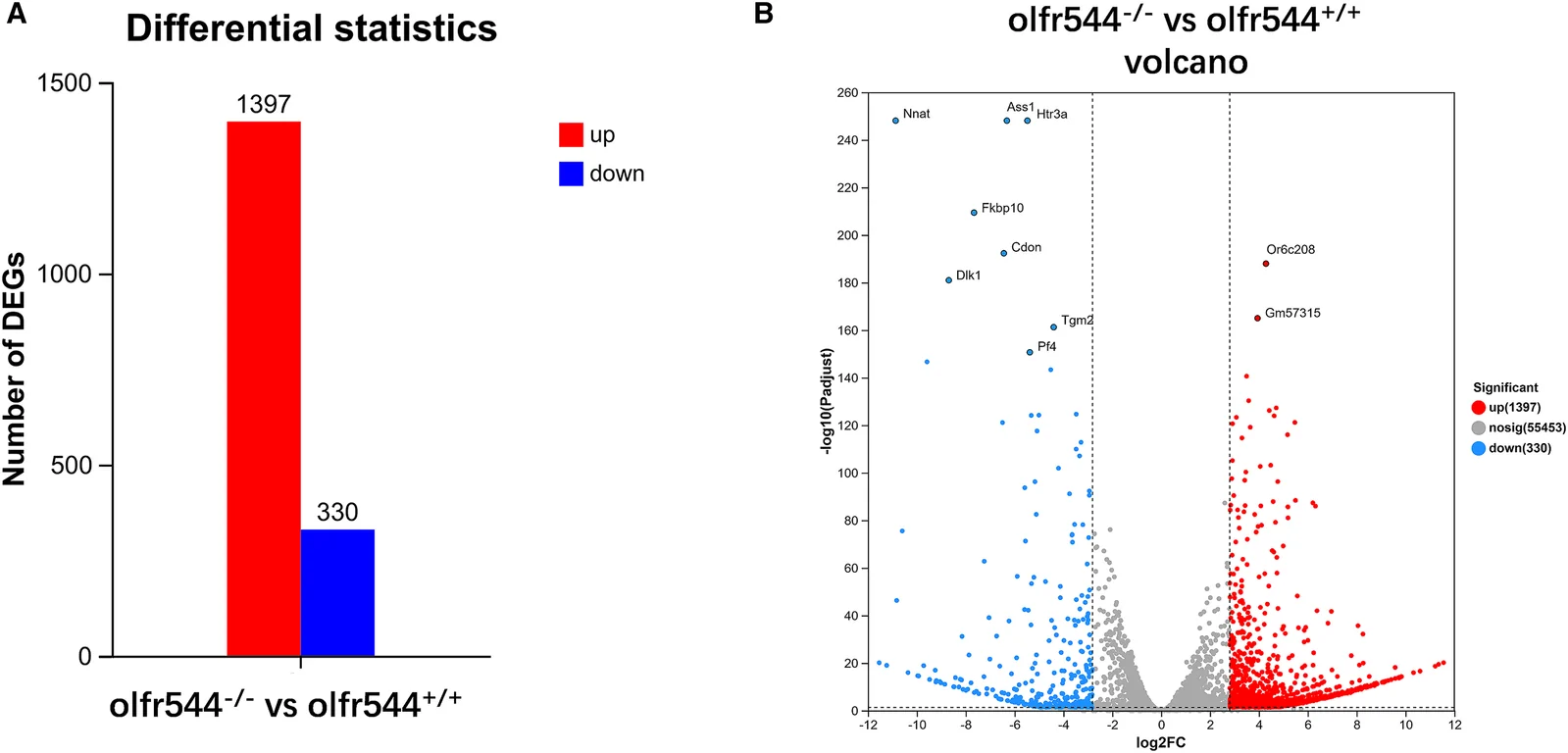
Differential gene expression analysis (A) Statistical analysis of expression differences between two groups. (B) Volcano plot showing differential gene expression between the two groups. RNA-seq data were obtained from *n* = 3 biologically independent N2a cell cultures per group. Differential expression analysis was performed with DESeq2 (absolute log2 fold change ≥2.807, *p*adj <0.05).

**Table 1:** Top 10 upregulated and downregulated genes in N2a *Olfr544*^−/−^ cells compared to N2a *Olfr544*^+/+^ cells

Upregulated gene	Differential expression (fold change)	Downregulated gene	Differential expression (fold change)
*Trap1a*	3021.264491	*Apbb1ip*	0.000336225
*Spag16*	2618.517734	*Mark4*	0.000415481
*Edil3*	2383.302668	*Nnat*	0.000536693
*Csmd3*	1548.804081	*Tead2*	0.000552495
*Grxcr2*	1286.125475	*Crabp1*	0.000647137
*Flt4*	914.8314119	*Slc7a8*	0.000761311
*Klrb1b*	881.303285	*Ubash3b*	0.001003051
*Galnt17*	872.3928441	*Gm57142*	0.001028447
*Rhox9*	869.1775323	*Igf2bp3*	0.001187165
*Gm45861*	851.9396463	*Acta2*	0.001306734

Italicized entries represent gene symbols.

Reactome enrichment analysis was performed on the 1,727 DEGs, with the majority associated with the cell cycle and nervous system (Figure 5A). Gene Ontology (GO) term analysis of *Olfr544*-deficient cells revealed significant enrichment for processes related to cellular communication, synaptic signaling, and regulation of biological processes (Figure 5B). To assess the molecular pathways in *Olfr544*-deficient N2a cells, Kyoto Encyclopedia of Genes and Genomes (KEGG) enrichment analysis was conducted separately on the upregulated and downregulated gene sets (Figures 5C and 5D). Following *Olfr544*-KO, the upregulated genes were predominantly enriched in the neuroactive ligand-receptor interaction pathway, involving 41 upregulated genes, including GPCRs and ion channel proteins, suggesting possible compensatory mechanisms in communication pathways. Conversely, the downregulated genes were primarily enriched in the motor protein pathway, involving 11 downregulated genes, such as myosin, actin, and kinesin. Similar results were obtained when Reactome analysis was applied to the upregulated and downregulated genes (Figures 5E and 5F). This downregulation of genes encoding myosin, actin, and kinesin family members is consistent with the observed morphological deficiencies in *Olfr544*-KO cells and aligns with the proposed role of Olfr544 in neurite dynamics.

**Figure 5:**
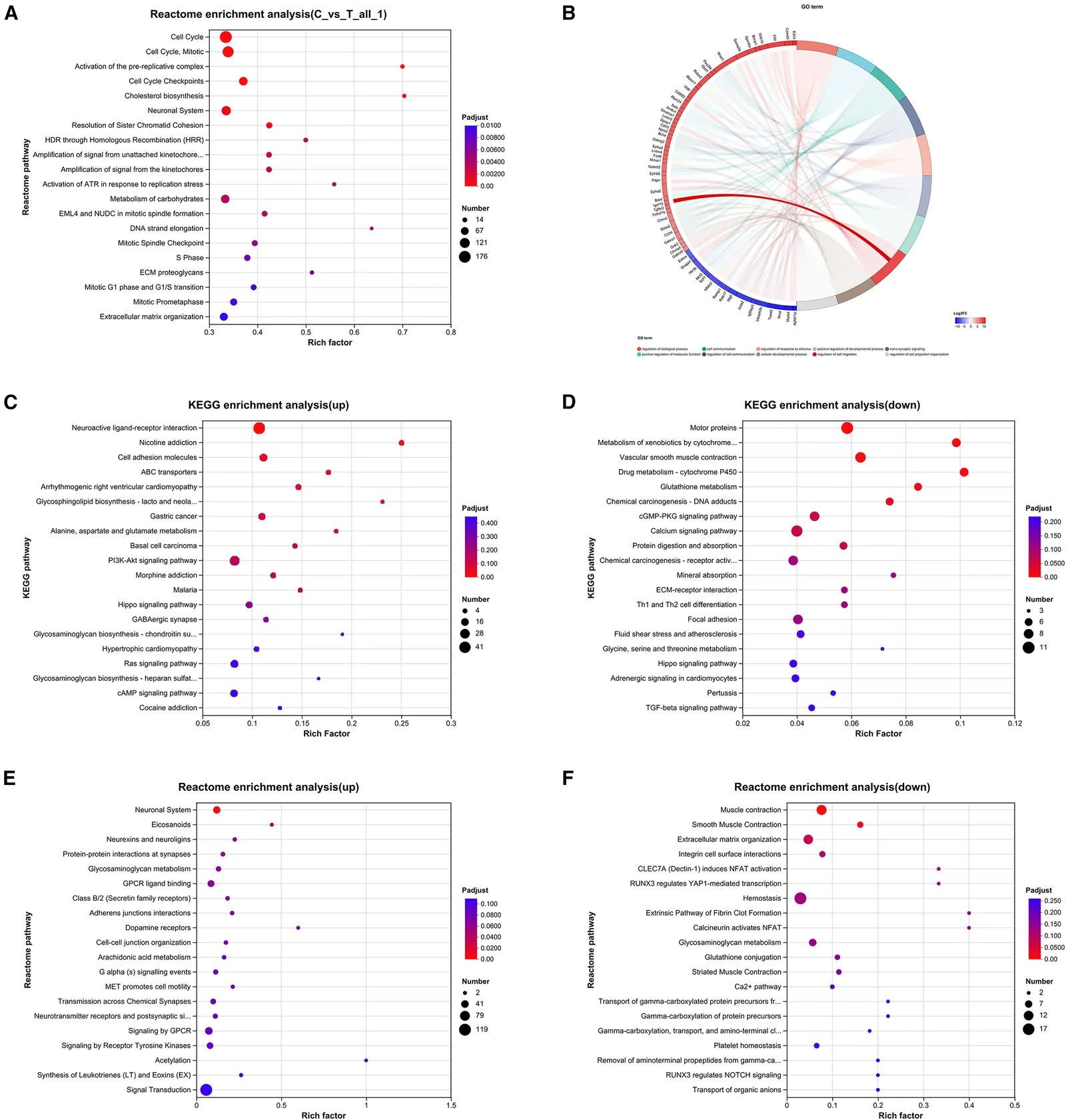
Functional enrichment analysis of differentially expressed genes (A) Reactome enrichment analysis of differentially expressed genes. (B) GO enrichment chord diagram of differentially expressed genes. (C) KEGG enrichment analysis of the upregulated gene set. (D) KEGG enrichment analysis of the downregulated gene set. (E) Reactome enrichment analysis of the upregulated gene set. (F) Reactome enrichment analysis of the downregulated gene set. RNA-seq data were obtained from *n* = 3 biologically independent N2a cell cultures per group. Functional enrichment analysis was performed with Reactome, GO, and KEGG databases.

### Identification of CREB-regulated candidate genes downstream of Olfr544

Given the central role of CREB activation in Olfr544 signaling, we sought to identify specific transcriptional targets of CREB that might mediate Olfr544’s effects. In our preliminary study, we examined the expression of CREB-regulated learning and memory-related proteins (Egr-1, c-fos, BDNF, and IGF-1) by western blot analysis, but no significant differences were detected. Consequently, we performed target gene prediction for the transcription factor CREB. Prediction analysis of transcription factor targets among the downregulated genes in *Olfr544*-KO cells identified three high-confidence candidates: *Igf2bp3*, *Kctd14*, and *Sorbs2* (Table 2). Because the RNA-seq data were derived from a baseline comparison between a monoclonal KO line and the parental bulk population, it is susceptible to clonal artifacts. Therefore, we utilized these sequencing results strictly as a hypothesis-generating screen and sought independent functional validation by investigating these targets following AZA treatment in the unedited parental WT clone. The WB (Western blot) results (Figures 6G–6J) corroborated the screening data. Crucially, IGF2BP3 exhibited the most robust regulation: independent of any CRISPR manipulation, its protein level was significantly elevated upon AZA treatment in WT cells. Furthermore, it was markedly reduced in *Olfr544*-KO cells, with no restoration by AZA (Figures 6G and 6H). This independent validation in the parent clone confirms IGF2BP3 as a key downstream effector whose expression is dynamically induced by Olfr544 signaling. The protein levels of SORBS2 and KCTD14 also showed a consistent decreasing trend in *Olfr544*-KO cells (Figures 6I and 6J). While not all pairwise comparisons reached statistical significance, the directional consistency across all three targets supports the conclusion that Olfr544 signaling contributes to maintaining their expression. Collectively, these protein-level data strengthen the link between Olfr544 activation and the regulation of specific effectors involved in neuronal function. Their identification as potential CREB targets provides a mechanistic link between Olfr544 activation, CREB phosphorylation, and the regulation of specific genes involved in neuronal structure and function.

**Table 2:** CREB target gene prediction results

TF name	Target gene ID	Target gene name	Start	Stop	Matched sequence	Cor	Cor *p* value	Cor *p*adjust
Creb3l2	ENSMUSG00000029814	*Igf2bp3*	255	263	GCCACGTGT	−0.91969	0.009415	0.131809
Creb3l2	ENSMUSG00000029814	*Igf2bp3*	508	516	GCCACGTGT	−0.91969	0.009415	0.131809
Creb3l2	ENSMUSG00000051727	*Kctd14*	498	506	GCCACGTGT	−0.9184	0.009717	0.045347
Creb3l2	ENSMUSG00000051727	*Kctd14*	516	524	GCCACGTGT	−0.9184	0.009717	0.045347
Creb3l2	ENSMUSG00000031626	*Sorbs2*	140	148	GCCACGTGT	−0.90657	0.012687	0.022203
Creb3l2	ENSMUSG00000031626	*Sorbs2*	156	164	GCCACGTGT	−0.90657	0.012687	0.022203
Creb3l2	ENSMUSG00000031626	*Sorbs2*	538	546	GCCACGTGT	−0.90657	0.012687	0.022203

TF name, transcription factor name; Target gene ID, target gene ID; Target gene name, target gene name; Start, start position (of TF binding site on target gene); Stop, stop position (of TF binding site on target gene); Matched sequence, matched sequence (TF binding site sequence); Cor, correlation coefficient (Pearson/Spearman correlation between TF gene and target gene expression, used to refine predictions; higher values suggest greater reliability); Cor *p* value, *p* value (significance of the correlation); Cor *p*adjust, adjusted *p* value (for the correlation). Italicized entries represent gene symbols.

**Figure 6:**
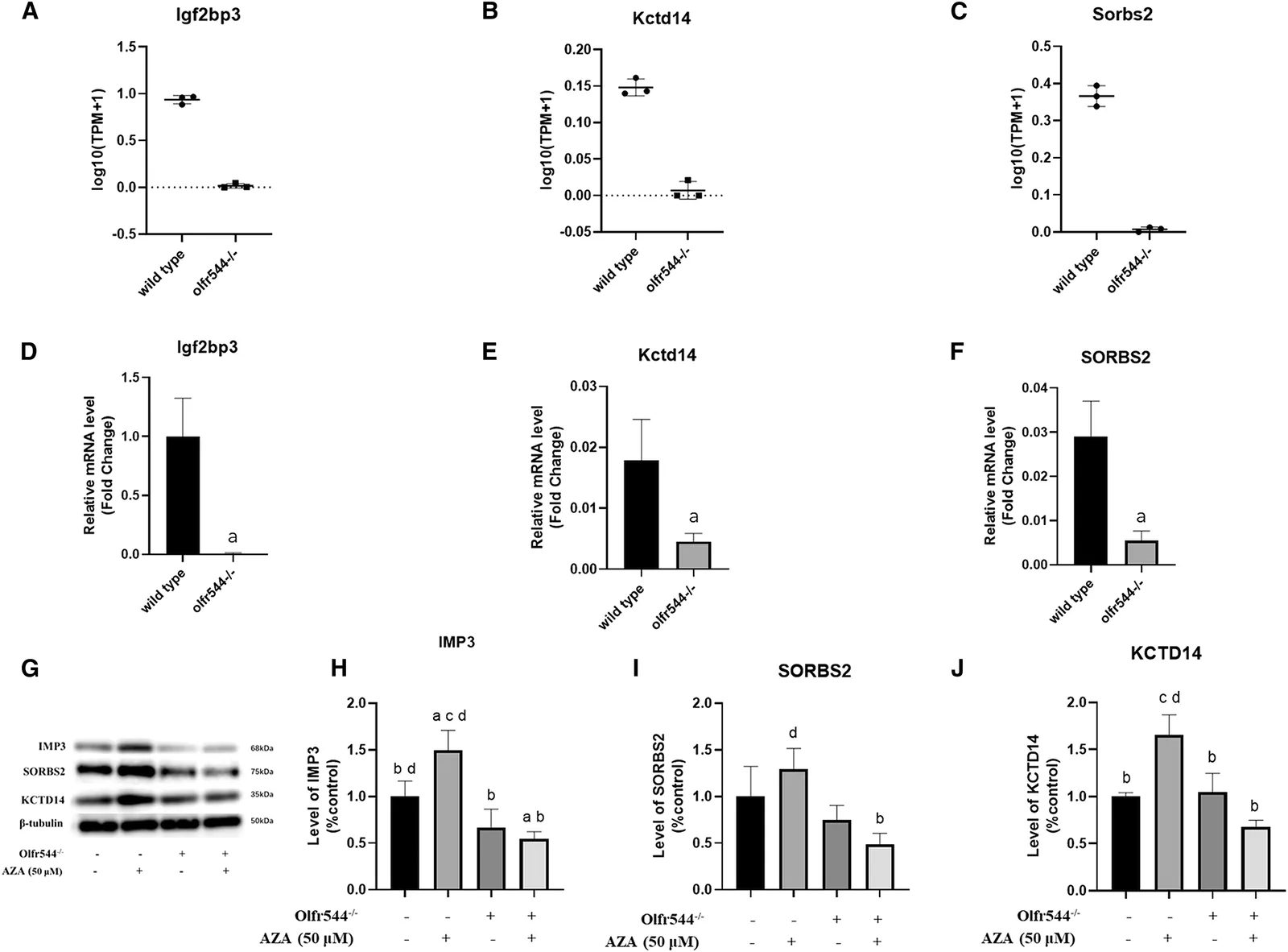
Expression and validation of CREB transcription factor target genes (A–C) Expression levels of CREB target genes obtained from RNA sequencing. (D–F) Validation of CREB target gene expression levels by RT-qPCR. (G–J) Western blot analysis of the corresponding proteins IGF2BP3, SORBS2, and KCTD14 in wild-type (WT) and *Olfr544*-knockout (KO) N2a cells, treated with or without AZA (50 μM, 24 h). Representative blot images are shown in (G), and quantification of protein levels normalized to β-tubulin is shown in (H) and (J). Data are presented as mean ± SEM from *n* = 3 biologically independent experiments. One-way ANOVA followed by Tukey’s HSD test and Student’s *t* test were performed for multiple group comparisons. (A) *p* < 0.05 compared to the control group (untreated WT cells); (B) *p* < 0.05 compared to WT + AZA; (C) *p* < 0.05 compared to KO cell; (D) *p* < 0.05 compared to KO + AZA. All experiments were performed in N2a cell cultures. For (G)–(J), cells were treated with AZA (50 μM, 24 h). Data are from *n* = 3 biologically independent experiments.

## Discussion

ORs account for approximately 60% of human GPCRs. Their widespread expression in non-olfactory tissues suggests functions beyond odor detection, and accumulating evidence links them to various diseases, including neurodegenerative conditions.^13^^,^^14^ In the brain, multiple ORs show altered expression in AD, Parkinson’s disease, and schizophrenia, often correlating with disease progression.^3^^,^^5^^,^^8^^,^^15^^,^^16^^,^^17^^,^^18^^,^^19^^,^^20^^,^^21^ Functional studies indicate that certain brain-expressed ORs can modulate microglial phagocytosis or tau phosphorylation,^22^^,^^23^^,^^24^ supporting their relevance as CNS GPCRs.^23^

Among extra-olfactory expressed ORs, Olfr544 is notable for its high expression near Aβ plaques in AD model mice.^8^ Its agonist AZA links it to metabolic sensing. Olfr544 regulates glucagon secretion, lipolysis, and mitochondrial biogenesis,^10^^,^^11^^,^^24^ but its brain function was unknown. Here, we confirmed endogenous expression of Olfr544 and its downstream effectors (*AC3* and *Gαolf*) in the N2a cell line. AZA promoted neurite outgrowth and significantly increased pCREB levels. In the nervous system, activated CREB is a central node linking extracellular signals to gene transcription critical for synaptic plasticity and neuronal survival. Our results demonstrate that AZA-induced CREB phosphorylation is strictly Olfr544-dependent, as it was completely abolished in KO cells. Thus, Olfr544 acts as an upstream initiator of the cAMP-PKA-CREB cascade in a neuronal context, mirroring canonical GPCR signaling pathways that govern morphological differentiation. However, unlike classical CREB activation that drives immediate-early genes or neurotrophins (e.g., c-fos and BDNF),^25^ we observed no significant changes in these targets (data not shown). This discrepancy suggests that Olfr544-mediated CREB activation may drive a more specialized transcriptional program in N2a cells, focused on cytoskeletal dynamics rather than generalized growth responses.

To further explore Olfr544-regulated pathways, we performed RNA-seq on *Olfr544*-KO N2a cells. We initially attempted gene silencing through RNAi but achieved poor knockdown efficiency due to low basal Olfr544 expression in N2a cells. RNA-seq revealed that upregulated genes were primarily enriched in the neuroactive ligand-receptor interaction pathway (41 genes, including GPCRs and ion channels), suggesting that the *Olfr544*-KO did not impair but potentially enhanced ligand-receptor interactions in neuronal cells, possibly due to cellular compensatory mechanisms. Downregulated genes were enriched in motor proteins (11 genes encoding myosin, actin, kinesin, etc.). Motor proteins are essential for neuronal function and morphogenesis,^26^^,^^27^^,^^28^ and their downregulation provides a molecular basis for the impaired adhesion and neurite outgrowth observed in *Olfr544*-deficient cells, underscoring the receptor’s critical role in maintaining structural neuronal homeostasis.

CREB target gene prediction identified three downregulated candidates: *Igf2bp3*, *Kctd14*, and *Sorbs2*, confirmed by qPCR and western blot. IMP3 (encoded by *Igf2bp3*), a key regulator of mRNA stability/transport critical for axon guidance and synaptic plasticity,^29^^,^^30^ showed the most pronounced and statistically robust Olfr544-dependent regulation: its protein level was elevated by AZA in WT cells and markedly reduced in KO cells, with no rescue. Given IGF2BP1 (a homolog) binds tau mRNA^31^ and IGF2BP3 variants correlate with tau burden in AD,^32^ Olfr544-CREB signaling may regulate neurite dynamics via modulating cytoskeletal protein mRNA metabolism. The protein levels of SORBS2 and KCTD14 followed a similar downward trend in the KO background, aligning with the transcriptional data. SORBS2, which maintains dendritic complexity and synaptic integrity,^33^^,^^34^^,^^35^^,^^36^ was significantly downregulated in KO cells, providing a direct link to impaired neuronal morphology. KCTD14, belonging to a neurodevelopmental disorder-associated family^37^^,^^38^^,^^39^^,^^40^ and phylogenetically related to KCTD7,^41^ showed consistent downregulation, suggesting potential roles in neuronal homeostasis that warrant further study. Although not all pairwise comparisons reached statistical significance, the consistent directional changes provide convergent evidence that Olfr544 signaling contributes to sustaining the expression of these proteins. The identification of *Igf2bp3*, *Kctd14*, and *Sorbs2* as candidate CREB targets provides a mechanistic link between Olfr544 activation and neuronal structure. Notably, IGF2BP3 showed the most robust regulation: elevated by AZA in WT cells and lost in KO cells (Figures 6G and 6H). This dual observation—upregulation by agonist in WT cells and loss of expression in KO cells—provides convergent evidence that IGF2BP3 expression is specifically dependent on Olfr544 signaling, while also circumventing the confounding effects of clonal variation that may influence baseline KO versus WT comparisons. SORBS2 and KCTD14 followed consistent downward trends, though their AZA responses were less pronounced. Collectively, these data strengthen the link between Olfr544-CREB signaling and effectors involved in mRNA metabolism and synaptic structure. While previous studies have shown that ORs can modulate microglial activity via diverse pathways, our findings establish a direct linear signaling axis from Olfr544 to the structural integrity of the neuron itself, supported by the significant enrichment of motor protein pathways in our RNA-seq data.

In summary, our *in vitro* findings demonstrate that Olfr544 activation by AZA initiates a cAMP-PKA-CREB cascade regulating cytoskeletal and motor protein-associated targets (IGF2BP3, SORBS2, and KCTD14). These results establish a direct mechanistic link between an extra-olfactory OR and neuronal structural plasticity. Although Olfr544 has been reported to be upregulated near Aβ plaques in AD model mice, the relevance of this pathway to AD remains speculative, as our study does not test *in vivo* function. To address this, future studies should: (1) employ *Olfr544*-KO or transgenic mouse models to assess causal roles in AD-related neuronal morphology and pathology; (2) identify endogenous brain ligands and confirm receptor activation *in vivo*; and (3) determine whether modulation of Olfr544 signaling influences disease-relevant outcomes such as synaptic integrity, neurodegeneration, or cognitive function.

### Limitations of the study

This study has several limitations. First, our primary conclusions are derived from the N2a neuroblastoma cell line. While this model is widely used for studying neurite outgrowth, it does not fully recapitulate the complexity of mature primary neurons or intact neural circuits. Second, the transcriptomic comparison between WT and *Olfr544*-KO cells was performed on a single monoclonal KO line; therefore, the DEGs should be interpreted as candidate pathways rather than definitive Olfr544-specific targets. Third, we did not perform rescue experiments by re-expressing Olfr544 in KO cells, which would provide additional genetic confirmation. Fourth, the functional link between CREB activation and the identified downstream targets (*Igf2bp3*, *Kctd14*, and *Sorbs2*) remains correlative; direct binding assays (e.g., chromatin immunoprecipitation [ChIP]) are needed to confirm CREB-mediated transcriptional regulation. Finally, direct protein-level confirmation of *Olfr544*-KO by western blot was not performed. While the KO was verified at the DNA and mRNA levels, and functional loss of signaling was inferred from abolished AZA-induced cAMP-PKA-CREB responses, we explicitly acknowledge that the presence or absence of Olfr544 protein itself remains unconfirmed due to the lack of a validated specific antibody. This represents a technical limitation of the study. Sixth, the N2a cell line is derived from a male mouse, and the sex of the HEK293T cell line is not definitively characterized. As only male-derived cell lines were used in this study, the potential influence of sex on the observed Olfr544-mediated signaling and morphological outcomes cannot be assessed. This represents a limitation to the generalizability of the findings, and future studies should include both male- and female-derived animal models to evaluate possible sex-dependent differences.

## Resource availability

### Lead contact

Requests for further information and resources should be directed to and will be fulfilled by the lead contact, Qian Chen (chenqian0731@outlook.com).

### Materials availability

• The *Olfr544* knockout N2a cell line generated in this study is available from the lead contact with a completed materials transfer agreement.
• The *Olfr544* expression plasmid, pME18S-Lucy-Flag-Rho, was a generous gift from Prof. Jennifer Pluznick (Johns Hopkins University) and is subject to the original materials transfer agreement; therefore, redistribution is restricted. Requests for this plasmid should be directed to Prof. Pluznick.
• Commercial antibodies, cell lines, and chemical agonists (e.g., AZA and forskolin) used in this study are available from the suppliers listed in the key resources table (see STAR Methods).

### Data and code availability

• Raw RNA-seq data have been deposited in the NCBI Sequence Read Archive under accession PRJNA1301537. Original uncropped western blot images are available at Mendeley Data (https://doi.org/10.17632/k5b36ssthj.1). All accession codes are listed in the key resources table. Microscopy data reported in this paper will be shared by the lead contact upon reasonable request.
• No custom code was generated for this study. All transcriptomic analyses were performed with publicly available software packages (fastp, HISAT2, StringTie, RSEM, and DESeq2) on the Majorbio Cloud Platform, as described in the STAR Methods.
• Any additional information required to re-analyze the data reported in this paper is available from the lead contact upon request.

## Acknowledgments

This work was supported by the 10.13039/501100001809National Natural Science Foundation of China (grant nos. 32060167 and 82260950) and Guizhou Science and Technology Department (grant no. ZK [2022]459).

## Author contributions

Conceptualization, S.L. and Q.C.; methodology, S.L. and Q.C.; validation, J.L., Y.Z., R.Y., and T.Z.; formal analysis, J.L., Y.Z., R.Y., and Y.D.; investigation, S.L., J.L., Y.Z., and Y.C.; resources, Q.C.; data curation, Y.D. and R.Y.; writing – original draft preparation, S.L. and J.L.; writing – review and editing, S.L., J.L., C.L., and Q.C.; visualization, S.L., J.L., Y.Z., R.Y., and T.Z.; supervision, C.L. and Q.C.; project administration, Q.C.; funding acquisition, T.Z. and Q.C. All authors have read and agreed to the published version of the manuscript.

## Declaration of interests

The authors declare no competing interests.

## Declaration of generative AI and AI-assisted technologies in the manuscript preparation process

During the preparation of this work, the authors used ChatGPT in order to polish language. After using this tool/service, the authors reviewed and edited the content as needed and take full responsibility for the content of the publication.

## STAR★Methods

### Key resources table

**Table:** 

REAGENT or RESOURCE	SOURCE	IDENTIFIER
**Antibodies**		

Anti-Flag	Santa Cruz Biotechnology	Cat# sc-166384, RRID:AB_2017592
Phospho-CREB (Ser133) Antibody	Cell Signaling Technology	Cat# 9191, RRID:AB_331606
CREB (48H2) Rabbit mAb	Cell Signaling Technology	Cat# 9197, RRID:AB_331277
Anti-IMP-3 Antibody [C2-E4]	Huabio	Cat# EM1701-15, RRID:AB_3068743
SORBS2 antibody	Proteintech	Cat# 24643-1-AP, RRID:AB_2879652
KCTD14 antibody	Proteintech	Cat# 21111-1-AP, RRID:AB_10733343
Beta Tubulin antibody	Proteintech	Cat# 66240-1-Ig, RRID:AB_2881629
Alexa Fluor 594 conjugated secondary antibody	Thermo Fisher Scientific	Cat# A-11005, RRID:AB_2534073
HRP-conjugated Goat Anti-Rabbit IgG(H + L)	Proteintech	Cat# SA00001-2, RRID:AB_2722564
HRP-conjugated Goat Anti-Mouse IgG(H + L)	Proteintech	Cat# SA00001-1, RRID:AB_2722565

**Chemicals, peptides, and recombinant proteins**		

Azelaic Acid	Sigma	246379-25G
Forskolin	Beyotime	S1612-1 mg
Dulbecco’s Modified Eagle Medium (DMEM)	Gibco	11960044
SQ22536	MCE	HY-100396
Lipofectamine 2000	Invitrogen	11668030
T4 DNA Ligase	Takara	2011A
NotI	Takara	1166S
EcoRI	Takara	1040S

**Critical commercial assays**		

MTT Cell Proliferation and Cytotoxicity Detection Kit	Beyotime	C0009S
Antifade Mounting Medium with DAPI	Beyotime	P0131-5 mL
PKA Kinase Activity Kit	Enzo	ADI-EKS-390A
Dual Luciferase Reporter Gene Assay Kit	Beyotime	RG027
Thermo Scientific RevertAid RT	Thermo Scientific	K1691
cAMP ELISA Detection Kit	Genscript	L00460

**Deposited data**		

RNA-seq of N2a cell	NCBI	PRJNA1301537
Original western blot images	mendeley data	https://doi.org/10.17632/k5b36ssthj.1

**Experimental models: Cell lines**		

Neuro-2a (N2a) cell	Procell	CL-0168
HEK293T cells	gift from Dr. Zhi Tang (Guizhou Medical University)	N/A

**Oligonucleotides**		

The RT-qPCR primers for the *olfr544*, *AC3* and *Gαolf* genes	Sangon	synthetic

**Recombinant DNA**		

pCREB-TA-Luc	Beyotime	D4050-1 μg
pME18S-Lucy-Flag-Rho	gift from Prof. Jennifer Pluznick (Johns Hopkins University)	JHU MTA A36291
Renilla expression vector	Beyotime	D2760-1 μg

### Experimental model and study participant details

The mouse neuroblastoma N2a cell line (Neuro-2a) was obtained from Procell (Cat# CL-0168). N2a cells originate from a male mouse (strain A, albino). HEK293T cells were a gift from Dr. Zhi Tang (Guizhou Medical University); the sex of this cell line is not definitively characterized. Both cell lines were cultured in Dulbecco’s Modified Eagle Medium (DMEM) supplemented with 10% fetal bovine serum (FBS) at 37°C in a humidified atmosphere with 5% CO_2_. Wild-type N2a cells carry endogenous Olfr544, while *Olfr544*-knockout N2a monoclonal clones were generated in-house via CRISPR-Cas9 editing as described in the CRISPR-Cas9 Knockout of Olfr544 subsection under STAR Methods. Cells were used at early passages to preserve cellular phenotype.

#### Sex-related considerations

Since only male-derived N2a and sex-undefined HEK293T cell lines were used in this study, we could not evaluate potential sex-dependent effects on the observed phenotypes. This limitation has also been noted in the limitations of the study section of the manuscript. No primary animal tissues, human participants, plants, or microbial strains were used in this work, so ethics committee approval was not required.

#### Cell line authentication and mycoplasma testing

The N2a cell line was authenticated by the supplier Procell. HEK293T cells were received as a laboratory gift. No independent STR-based genotypic authentication was performed in our laboratory. We routinely monitored cell identity by morphology and expected functional performance, but we recognize that this monitoring cannot serve as definitive genotypic authentication and note this point as a limitation of our study. Routine in-house mycoplasma testing was not performed throughout this study, which is also acknowledged as a limitation.

#### Ethics statement

This study exclusively used commercially available or donated cell lines and did not involve animals, human participants, or human-derived samples. As such, institutional ethics committee approval was not required.

### Method details

#### Cell culture

HEK293T cells (a gift from Dr. Zhi Tang’s laboratory) and N2a cells were cultured in Dulbecco’s Modified Eagle Medium (DMEM) supplemented with 10% fetal bovine serum (FBS). The cells were incubated in a 5% carbon dioxide environment at 37°C. For differentiation, N2a cells were treated with 500 μM AZA in serum-deprived DMEM medium for 24 h. For morphological assessment, the percentage of cells exhibiting neurite outgrowth was quantified using phase-contrast microscopy. Neurites were defined as a process with lengths equivalent to one or more diameters of a cell body. For each sample, images were captured from 10 randomly selected fields of view, and the experiment was repeated in three independent biological replicates. For Western blot analysis, N2a cells were serum-starved overnight and then treated with 50 μM AZA for either 30 min (for pCREB/CREB detection) or 24 h (for other targets). All Western blot experiments were also performed with three independent biological replicates.

#### Reverse transcription-polymerase chain reaction

Total RNA was extracted from N2a cells using a commercial kit and treated with DNase I to eliminate genomic DNA contamination, following the manufacturer’s instructions. The extracted RNA served as the template for first-strand cDNA synthesis via reverse transcription, using Oligo(dT) primers and reverse transcriptase. This cDNA was then used as the template for PCR amplification targeting the olfactory receptor gene *Olfr544* and its downstream molecules, Golf and AC3. For Golf and AC3, the primers were designed to span at least one intron, thereby preventing false-positive amplification from potential genomic DNA contamination; however, such design was not applicable for *Olfr544* as this gene lacks introns. The PCR reactions were run for 30 cycles. Primer sequences and expected product sizes are detailed in Table S1. The resulting PCR products were analyzed by agarose gel electrophoresis and confirmed by DNA sequencing.

#### Cell viability assay

Cell viability was assessed using the MTT Cell Proliferation and Cytotoxicity Assay Kit, following the manufacturer’s instructions. N2a cells were seeded into 96-well plates at a density of 5 × 10^3^ cells/well in DMEM (*n* = 6 per group). After 24 h of treatment with different concentrations of AZA, 10 μL of MTT solution was added to each well, and the plates were incubated for 4 h at 37°C. Subsequently, 100 μL of dimethyl sulfoxide (DMSO) was added to solubilize the formazan crystals. The absorbance was measured at 570 nm using a Thermo Scientific Multiskan FC microplate reader (Thermo Fisher Scientific). Each experiment was performed in six biologically independent replicates.

#### CRISPR-Cas9 knockout of *Olfr544*

Two sgRNA sequences (sgRNA1: GGGAGTAGGAGGCACCTAAC; sgRNA2: GGGAGGCAGTGCGCGCCACA) targeting exon 2 of the mouse *Olfr544* gene were designed and constructed into an sgRNA vector containing the spCas9 protein gene. Approximately 1×10^6^ cells were seeded into petri dishes and divided into experimental and control groups. The experimental group was transfected with 2 μg of endotoxin-free plasmid, while the control group received no transfection. Seventy-two h post-transfection, puromycin was added to both groups at a final concentration of 4 μg/mL to screen for positive cells. After 72 h of antibiotic selection, the control group cells were essentially dead, whereas surviving cells in the experimental group were expanded. Genomic DNA was extracted from polyclonal pools and subjected to PCR amplification using primers flanking the target site (PCR F: CAGAGACGAATGCCCACTTGAA; PCR R: CCGCTACCCAGAAATAGTGACA). Sanger sequencing of the PCR products confirmed the introduction of a 149 bp deletion at the target site, which is not a multiple of three and is predicted to cause a frameshift and premature stop codon. Cells carrying the deletion were subsequently subjected to limited dilution to isolate monoclonal populations. Genomic DNA from individual clones was again amplified and sequenced to verify the uniform knockout allele (Figure S2). To confirm loss of *Olfr544* expression at the transcript level, total RNA was extracted from the monoclonal knockout cells and analyzed by RT PCR, which showed absence of the wild type *Olfr544* mRNA (Tables S2), consistent with nonsense mediated decay of the mutated transcript. However, we acknowledge that direct protein-level confirmation of *Olfr544* loss is lacking due to the unavailability of a validated specific antibody.

#### Measurements of cAMP and PKA activity assay in N2a cells

Intracellular cAMP concentrations and PKA kinase activity were measured using the cAMP ELISA Detection Kit and PKA Kinase Activity Kit, respectively, according to the manufacturers’ protocols. Briefly, N2a wild-type and gene-knockout cells were treated with 50 μM AZA or 1 μM FSK for 30 min, with each condition performed in three independent biological replicates. For cAMP detection, culture supernatants were collected, centrifuged at 1,000 × g for 10 min, and diluted 2-fold. cAMP standards were prepared in serum-free medium, and samples were assayed in duplicate. Absorbance was measured at 450 nm, and concentrations were calculated by interpolation from the standard curve. For PKA activity, cells were lysed and clarified supernatants were incubated in a pre-coated substrate plate with ATP at 30°C. Phosphorylated substrate was detected using a phosphospecific primary antibody followed by an HRP-conjugated secondary antibody and TMB development. Absorbance was read at 450 nm, and activity was normalized to total protein concentration with reference to a purified PKA standard.

#### Protein extraction and immunoblotting

Total protein was extracted from cell lysates using preheated SDS sample buffer (100 μL). The lysates (10 μL) were resolved on 10%–12% SDS-polyacrylamide gels and electrophoretically transferred to PVDF membranes (Millipore). Membranes were blocked with 5% nonfat milk in TBST (Tris-buffered saline with 0.1% Tween 20) for 1 h at room temperature, followed by overnight incubation at 4°C with primary antibodies. After three washes with TBST, membranes were incubated with appropriate HRP-conjugated secondary antibodies (Proteintech) for 1 h at room temperature. Protein bands were detected using an ECL substrate (Bio-Rad) and visualized with a ChemiDoc™ MP imaging system (Bio-Rad). Band intensities were quantified using ImageJ software. Data analysis and visualization were performed using GraphPad Prism 8. Results represent at least three biologically independent experiments.

#### Immunocytochemistry

HEK293T cells were seeded into 24-well plates and cultured for 24 h. Transfection was performed using Lipofectamine 2000 with the pME18S-Lucy-Flag-Rho-*Olfr544* plasmid, followed by an additional 24 h of incubation. Cells were then washed twice with PBS and fixed with 4% paraformaldehyde for 10 min at room temperature. After fixation, cells were permeabilized and blocked with 5% bovine serum albumin (BSA) in PBS for 45 min, then incubated overnight at 4°C with an anti-Flag primary antibody (diluted 1:200 in blocking buffer). Following washes, cells were incubated for 1 h in the dark with an Alexa Fluor 594-conjugated secondary antibody (1:1000, Thermo Fisher Scientific). After final washes, nuclei were stained with DAPI. Images were acquired directly from the plate using a Leica SP8 confocal microscope equipped with a suitable objective for plate-based imaging. In the acquired images, the Flag-tagged *Olfr544* fusion protein was visualized in red, and nuclei were stained blue with DAPI.

#### Cloning of pME18S-lucy-flag-Rho-*Olfr544* and CRE-luciferase assay

To construct the pME18S-Lucy-Flag-Rho-*Olfr544* plasmid, the pME18S-Lucy-Flag-Rho vector was first obtained from Dr. Jeniffer Pluznick (Johns Hopkins University, USA). The full-length cDNA of *Olfr544* was then inserted into this vector using EcoRI and NotI restriction enzymes (Takara, Beijing, China). The resulting recombinant plasmid was co-transfected with the pCREB-TA-Luc reporter plasmid and the internal control Renilla luciferase expression vector pRL-TK into HEK293T cells using Lipofectamine 2000 (Invitrogen). At 24 h post-transfection, cells were treated with various concentrations of AzA for 24 h. For the adenylyl cyclase inhibitor experiment, cells were pre-treated with 200 μM SQ22536 for 18 h, followed by treatment with different concentrations of AzA for an additional 24 h in the continued presence of SQ22536. Luciferase activity was measured using a dual-luciferase assay kit, and luminescence was quantified with a Feyond-A300 chemiluminescence detector (Allsheng, China). Firefly luciferase signals were normalized to the corresponding Renilla luciferase signals.

#### RNA extraction and transcriptome sequencing

RNA purification, reverse transcription, library construction, and sequencing were performed by Shanghai Majorbio Bio-pharm Technology Co., Ltd. using an established protocol with modifications[11]. Polyadenylated mRNA was isolated via oligo(dT) magnetic bead enrichment. Library quality control and quantification were performed using an Agilent 5300 Bioanalyzer and a Nanodrop 2000 spectrophotometer. The sequencing library was prepared on the NovaSeq X Plus platform (PE150) using the NovaSeq Reagent Kit.

Raw paired-end sequencing reads underwent quality trimming and filtering using fastp with default parameters[12]. Subsequently, reference-guided alignment of clean reads was performed in orientation-aware mode via HISAT2[13]. Transcript assembly for individual samples was conducted using StringTie under a reference-based framework[14]. Functional annotation of assembled transcripts involved alignment against six major databases: NCBI non-redundant protein (NR), Swiss-Prot, PFAM, Clusters of Orthologous Groups (COG), Gene Ontology (GO), and Kyoto Encyclopedia of Genes and Genomes (KEGG).

#### Differential expression analysis

Transcriptomic analyses were conducted on the Majorbio Cloud Platform (www.majorbio.com). Gene expression quantification was performed using RSEM (v1.3.1) with transcripts per million (TPM) as the normalization metric[15, 16]. Differentially expressed genes (DEGs) were identified using DESeq2 (v1.24.0) with thresholds of false discovery rate (FDR) < 0.05 and |log2 fold change| > 1[17]. Functional enrichment analysis of DEGs was carried out against Kyoto Encyclopedia of Genes and Genomes (KEGG) pathways using KOBAS, with statistical significance defined by Fisher’s exact test (corrected P-value <0.05)[18]. Visualization of results was achieved using a customized D3.js implementation (v7.0.0) by Shanghai Majorbio Bio-pharm Technology Co., Ltd.

#### Transcription factor–target predict

The prediction of transcription factor CREB (cAMP response element-binding protein) target genes among differentially expressed genes (DEGs) showing significant downregulation in the *Olfr544*-KO group was performed using the Majorbio Cloud Platform.

### Quantification and statistical analysis

All quantitative data are presented as mean ± SEM, with the exact sample size (n) detailed in figure legends, where n represents the number of biologically independent experiments. Statistical comparisons between two groups were performed using unpaired two-tailed Student’s t-tests. For multiple group comparisons, one-way ANOVA followed by Tukey’s post hoc test was applied. A *p*-value <0.05 was considered statistically significant. Transcriptomic data were analyzed on the Majorbio Cloud Platform: gene expression was quantified using RSEM, differential expression analysis was performed with DESeq2 (thresholds: padj <0.05 and |log_2_FC| > 1), and functional enrichment was assessed using Fisher’s exact test. All obtained data were included unless invalidated by technical failure.
