# Supplementary material for: Role of olfactory receptor Olfr544 in neuronal cells: Insights into neurite outgrowth and CREB signaling
Source: iScience. 2026 Oct 9;29(11):117757. doi: 10.1016/j.isci.2026.117757 (PMC13677197; doi:10.1016/j.isci.2026.117757)
Supplement: Supplementary file 1 [file mmc1.pdf]

iScience, Volume *29*

Supplemental information

Role of olfactory receptor Olfr544 in neuronal cells: Insights into neurite outgrowth and CREB signaling

Shuhan Lv, Jia Liu, Yiyuan Zhou, Rong Yang, Taiwei Zhang, Yunyan Deng, Yan Chen, Chencen Lai, and Qian Chen

Supplemental Information

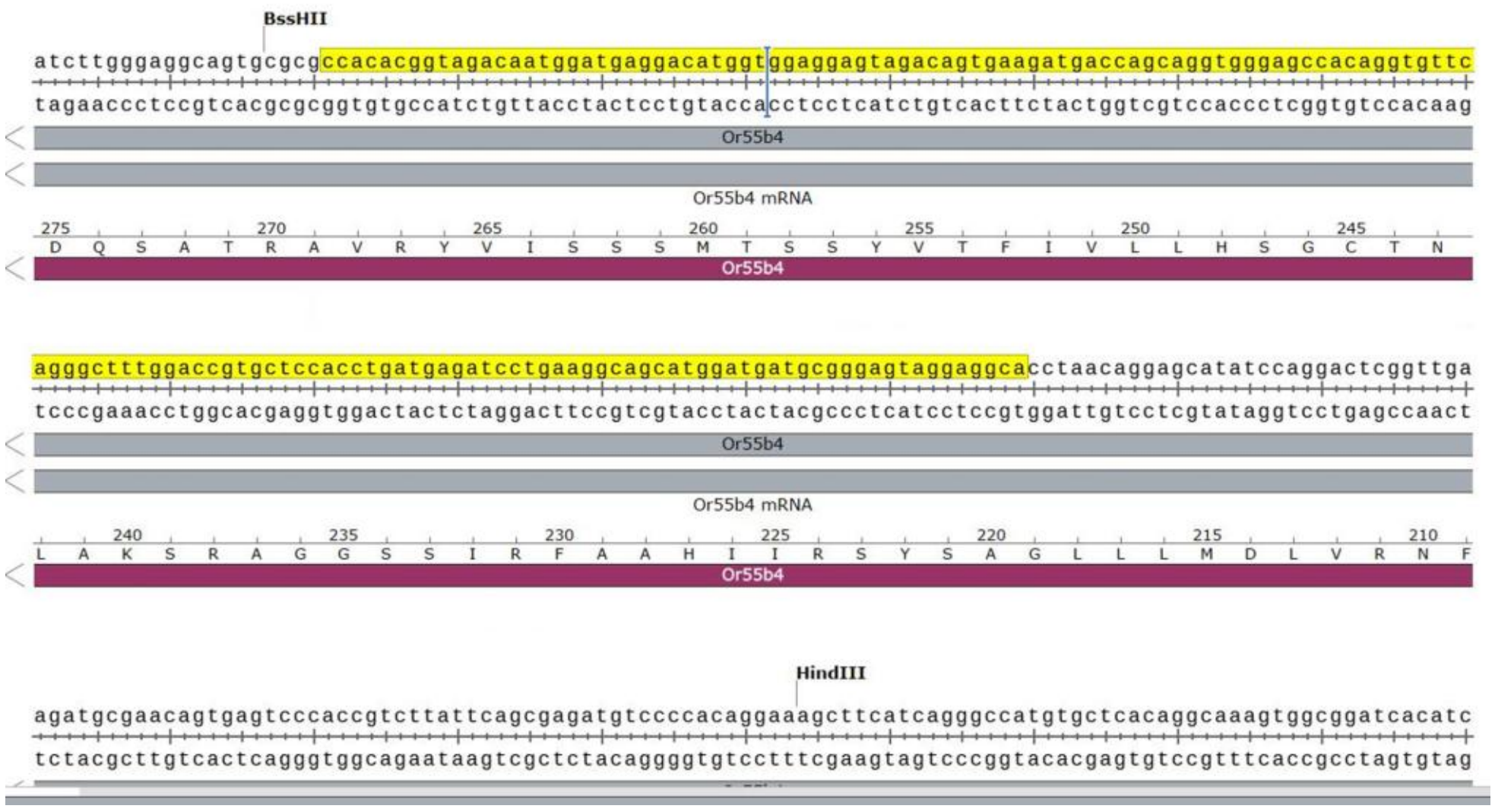

Figure S1. Schematic representation of sgRNA design targeting exon 2 of the mouse *Olfr544* gene. The region highlighted in yellow indicates the 149-bp genomic sequence deleted in the knockout clone. The deletion causes a frameshift, predicted to result in a premature stop codon and loss of functional *Olfr544* protein.

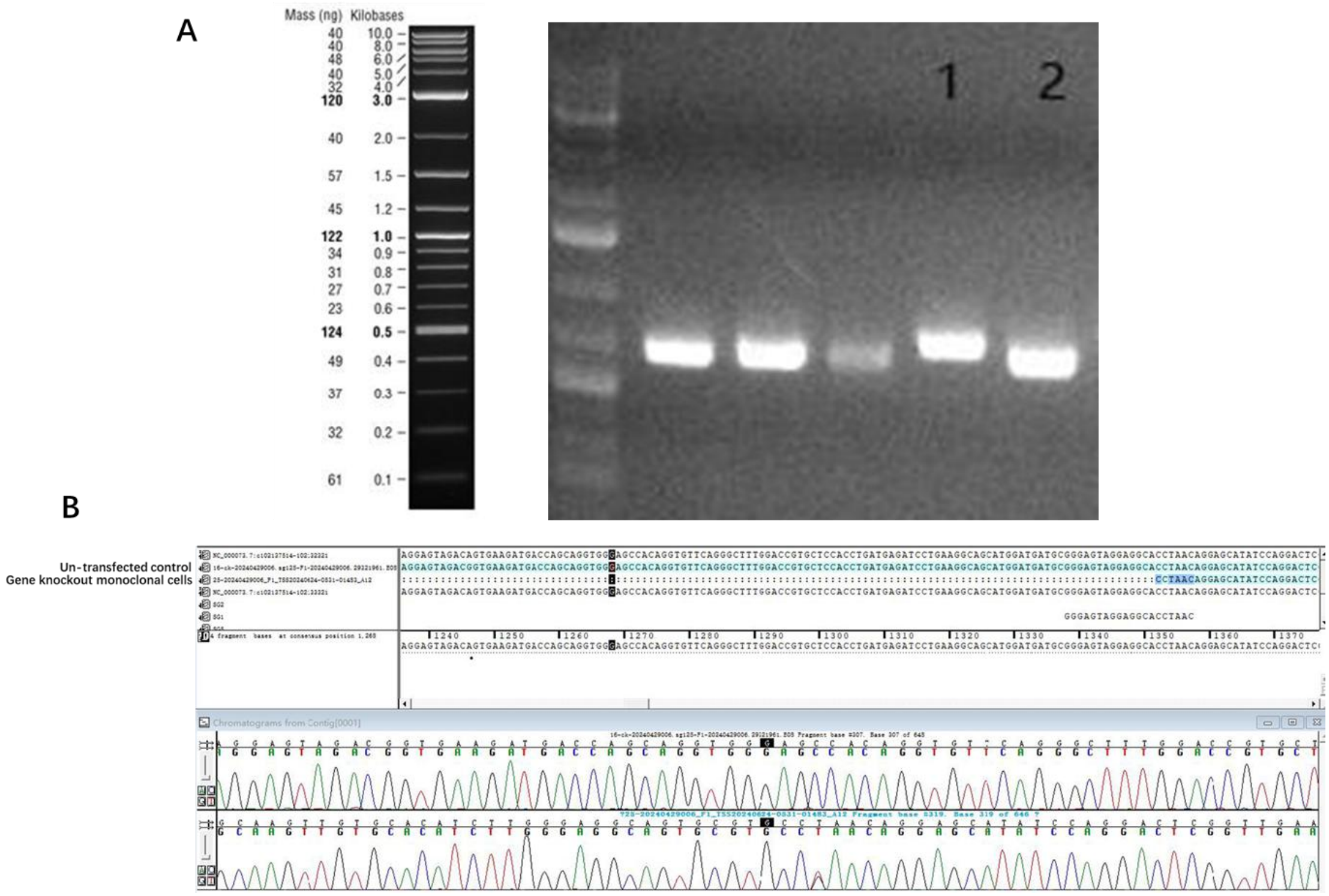

Figure S2. Genomic validation of *Olfr544* knockout monoclonal N2a cell clones. (A) Agarose gel electrophoresis of genomic PCR products from monoclonal cells. Lane 1 represents the un-transfected control group cells, while Lane 2 contains the gene-knockout monoclonal cells. The expected size of the wild-type control amplicon is 687 bp. (B) Sanger sequencing analysis comparing *Olfr544*-knockout monoclonal cells versus control cells.

Table S1. RT-PCR primer sequences for *Olfr544*, *AC3* and *Gnal*

Gene Name	Primers	Product Length	Spanning Introns
olfr544	Forward 5'-CTTTACTTCCCCTGCCTGTGT-3'<br>Reverse 5'-CAATGCATGTGCTCCTGGTG-3'	642bp	NO
AC3	Forward 5'-CATTGACCCCTGGCTGATGA-3'<br>Reverse 5'-AGTACTTGGAAGGCACCAGG-3'	569bp	YES
Gnal	Forward 5'-CCTGGGACGTTCGAAGGATG-3'<br>Reverse 5'-CTGCTTCTTTTCCTCGGGGT-3'	614bp	YES

Table S2. qRT-PCR validation of *Olfr544* transcript levels in wild-type and *Olfr544*-knockout N2a cells.

Normalized expression value[1]		log2(fold change)[2]
WT N2a cell (std)	*Olfr544*-KO cell (std)	
0.148 (0.035)	0.011 (0.004)	3.76*

[1] Normalized expression value as $2^{\Delta CT}$, with the expression of the Beta Tubulin gene used to normalize values. Normalized expression values for WT N2a cell and *Olfr544*-KO cell were obtained as an average (±standard deviation) over three biological samples.

[2] Average fold changes as log2 (normalized expression value of WT N2a cell /normalized expression value *Olfr544*-KO cell). The significance of the difference in expression value between WT N2a cell and *Olfr544*-KO cell was calculated using the t-test. *P <0.05.
